# A 28-day, randomized, double-blind, placebo-controlled, parallel group study of nebulized revefenacin in patients with chronic obstructive pulmonary disease

**DOI:** 10.1186/s12931-017-0647-1

**Published:** 2017-11-02

**Authors:** Krishna K. Pudi, Chris N. Barnes, Edmund J. Moran, Brett Haumann, Edward Kerwin

**Affiliations:** 1 Upstate Pharmaceutical Research, Greenville, SC USA; 2 Theravance Biopharma US, Inc., South San Francisco, CA USA; 3 Theravance Biopharma UK Limited, London, UK; 4 Clinical Research Institute of Southern Oregon, PC, Medford, OR USA

## Abstract

**Background:**

Revefenacin is a once-daily long-acting muscarinic antagonist (LAMA) in clinical development for the treatment of patients with chronic obstructive pulmonary disease (COPD). In a dose-ranging study, nebulized once-daily revefenacin had a long duration of action in patients after 7 days’ administration of doses up to 700 μg. In this multiple-dose study, the bronchodilation efficacy and adverse events profile were characterized in patients administered nebulized revefenacin once daily for 28 days.

**Methods:**

A total of 355 COPD patients (mean age 62 years, mean forced expiratory volume in 1 s [FEV_1_] 44% of predicted) were randomized in a double-blind, placebo-controlled parallel group study. Inhaled corticosteroids as well as short-acting bronchodilators were permitted. Once-daily treatments (44, 88, 175 or 350 μg revefenacin or matching placebo) were administered by a standard jet nebulizer, for 28 days. The primary endpoint was change from baseline in D28 trough FEV_1_, and secondary endpoints included weighted mean FEV_1_ over 0 to 24 h and rescue medication (albuterol) use. Safety evaluations included adverse events, laboratory assessments, electrocardiograms and 24-h Holter profiles.

**Results:**

Revefenacin (88, 175 and 350 μg) significantly improved D28 trough FEV_1_ over placebo (187.4, 166.6 and 170.6 mL, respectively, all *p* < 0.001); 44 μg produced a sub-therapeutic response. At doses ≥88 μg, more than 80% of patients achieved at least a 100-mL increase from baseline FEV_1_ in the first 4 h post dose compared with 33% of placebo patients. For doses ≥88 μg, D28 24 h weighted mean differences from placebo for FEV_1_ were numerically similar to respective trough FEV_1_ values, indicating bronchodilation was sustained for 24 h post dose. Doses ≥88 μg reduced the average number of albuterol puffs/day by more than one puff/day. The 350 μg dose did not demonstrate additional efficacy over that observed with 175 μg revefenacin. Revefenacin was generally well tolerated, with minimal reports of systemic anti-cholinergic effects.

**Conclusions:**

These data suggest that 88 and 175 μg revefenacin are appropriate doses for use in longer-term safety and efficacy trials. Revefenacin offers the potential for the first once-daily LAMA for nebulization in patients with COPD who require or prefer a nebulized drug delivery option.

**Trial registration:**

ClinicalTrials.gov NCT02040792. Registered January 16, 2014.

## Background

Long-acting inhaled bronchodilators are recommended as maintenance therapy for chronic obstructive pulmonary disease (COPD) patients with moderate to severe symptoms or those who are at higher risk for COPD exacerbations [1]. These bronchodilators fall into two classes: long-acting muscarinic antagonists (LAMAs) and long-acting beta agonists (LABAs), with the once-daily LAMA, tiotropium, and the twice-daily LABAs, salmeterol and formoterol, being the most widely prescribed for COPD [2–4]. Moreover, monotherapy with a LAMA is considered a first-line therapy option for many patients with moderate to severe COPD [1, 5].

Long-acting inhaled bronchodilators are most often self-administered with hand-held devices such as pressurized metered-dose inhalers or dry powder inhalers [6, 7]. Some patients, however, have difficulty using hand-held devices (eg, the elderly or cognitively impaired) [8, 9], which can result in inaccurate dosing, poor adherence and potentially poor clinical outcomes [10–12]. Nebulizers, which are easy to use and offer similar efficacy [8, 13], are an alternative to hand-held devices. Yet there are limited options for maintenance treatment with nebulized long-acting bronchodilators. Nebulized LABAs (Brovana® [arformoterol tartrate] and Perforomist® [formoterol fumarate]) are currently available; however, these agents are labelled for twice-daily administration. Thus, despite the ascendency of effective and convenient once-daily LAMA bronchodilator therapy (tiotropium bromide) in clinical practice for both reversal of bronchoconstriction and control of acute exacerbations of COPD, and the recent emergence of additional once-daily LAMA, LABA/ICS and LAMA/LABA handheld products, no once-daily nebulized bronchodilator has been available to patients who require or prefer nebulized drug delivery [9]. Revefenacin is the first once-daily anti-muscarinic administered via a standard jet nebulizer to enter late-stage clinical development [9, 14, 15].

Research has shown that revefenacin may produce sustained, long-acting bronchodilation similar to tiotropium bromide but with a lower potential for anti-muscarinic side effects (eg, dry mouth) [16]. In a randomized, double blind, repeat dose, placebo-controlled dose-ranging study, nebulized revefenacin (22 to 700 μg administered for 7 days via a standard jet nebulizer) demonstrated a long duration of action and low systemic exposure in patients with COPD [17]. Moreover, doses 88 μg and above produced clinically effective bronchodilation as measured by trough forced expiratory volume in 1 s (FEV_1_) and serial spirometeric assessments following the D7 dose [17]. The objective of this 28-day study was to identify appropriate revefenacin doses for longer-term safety and efficacy trials in patients with COPD.^1^

## Methods

### Study design and conduct

This randomized, double-blind, placebo-controlled, multiple-dose, parallel-group study, conducted at 41 centers in the United States between April 2014 and July 2014, evaluated the efficacy, safety and tolerability of revefenacin in patients with COPD (ClinicalTrials.gov NCT02040792). The study conformed to appropriate ethical guidelines and was conducted in accordance with the principles of the International Conference on Harmonisation of Technical Requirements for Registration of Pharmaceuticals for Human Use guideline for good clinical practice [18] and the code of ethics of the World Medical Association’s Declaration of Helsinki [19], with all patients providing written informed consent.

### Patients and treatments

Patients were men or women of non-childbearing potential (aged 40 to 75 years) with moderate to severe COPD [1] and a current or former smoking history of more than 10 pack-years, who at screening demonstrated a post-ipratropium bromide ratio of FEV_1_/forced vital capacity of <0.7 and a FEV_1_ of 30–80% of the predicted normal value after withholding short-acting bronchodilators for ≥6 h and long-acting bronchodilators for ≥14 days. In addition, patients were eligible for enrollment regardless of the numerical improvement in their FEV_1_ following bronchodilator (ipratropium bromide and/or albuterol) administration via a PARI LC^®^ Sprint nebulizer (PARI, Midlothian, Virginia, US) at screening. Patients who demonstrated reversiblity to placebo (≥12% change and ≥200 mL) were excluded from the study.

Following the screening period (two or three visits no more than 50 days prior to D1 of the treatment period), patients were stratified at randomization based on responsiveness status to ipratropium bromide during screening (responsive or non-responsive, with responsiveness defined as ≥12% and ≥200 mL change in FEV_1_ 45 min after completion of ipratropium bromide nebulization). Randomized patients received a 3-mL inhalation solution of placebo or revefenacin (44, 88, 175 or 350 μg) administered once daily for 28 days from a PARI LC Sprint nebulizer. Dosing occurred in the morning and was self-administered, except on D15, D16 and D28, when patients were dosed at their clinical study center.

All study personnel, as well as the sponsor, site monitors and patients, were blind to treatment allocation throughout the study.

Concomitant therapy was managed during the study as follows: LABA and LAMA bronchodilators were prohibited; combination steroid and LABA therapy was discontinued and the steroid component was replaced with an equivalent strength steroid inhaler monotherapy. Albuterol was allowed as necessary but was requested to be withheld for 6 h before the initial spirometry assessment performed at each study visit and during the course of the entire visit when serial spirometry was performed (unless medically necessary).

### Assessments and endpoints

For FEV_1_ assessment, spirometry was performed according to techniques consistent with the American Thoracic Society guidelines for spirometry [20] and conducted at specified time points during the screening visits and treatment period (D1 baseline [45 min and 15 min predose; 0 to 6 h postdose]; D15, D16 and D28 pretreatment trough [45 min and 15 min predose]; and D28/29 post treatment [0 to 24 h]). Peak expiratory flow rate (PEF) was conducted by the participants each morning and evening throughout the treatment period and up to the post-treatment follow-up visit. Safety and tolerability were assessed based on adverse events (AEs), clinical laboratory findings, vital signs, physical examinations and/or electrocardiogram (ECG) readings obtained before, during and after the treament period. A diary was used for recording dosing times, rescue medication use, concomitant medications and AEs throughout the 28-day dosing period and the 1-week follow-up period.

The primary endpoint was change from baseline in D28 trough FEV_1_ (the mean of the 23.25-h and 23.75-h time points following the last dose on D28) measured on D29. Secondary endpoints included the D1 change from baseline of the weighted mean FEV_1_ over 0 to 6 h (FEV_1(0–6h)_), and the D28 change from baseline of the weighted mean FEV_1_ over 0 to 12 h (FEV_1(0–12h)_) and 0 to 24 h (FEV_1(0–24h)_) (serial spirometry); time to a 100-mL increase from baseline on D1; change in PEF and use of albuterol rescue medication (puffs per day and percentage of rescue medication–free days).

Pharmacokinetic (PK) blood samples were obtained on D28 before dosing and post dose (at 0.25, 0.5, 1, 2, 4, 6, 8, 12, 24, 48, 72 and 96 h) at selected sites and in 45 subjects. Concentrations of revefenacin were quantified using validated liquid chromatography with tandem mass spectrometry (lower limit of quantification of 0.005 ng/mL). The analytical method for revefenacin was validated in K2-EDTA (ethylenediaminetetraacetic acid) plasma, utilizing solid phase extraction. Revefenacin concentrations were calculated using a standard curve with a 1/x^2^ linear regression over a concentration range of 0.005 to 2.5 ng/mL. The mass spectrometer (API-5000™ System [Applied Biosystems, Ontario, Canada]) was operated under optimized conditions for the detection of revefenacin in turbo ion spray, positive ionization and selected reaction monitoring.

### Statistical analysis

Based on a sample size of 350 (70 participants assigned to each treatment group) and allowing for 45 dropouts, 60 completers in each group provided at least 80% power to detect an increase of 120 mL in trough FEV_1_, assuming a standard deviation of 200 mL and a 5% significance level.

The intent-to-treat (ITT) population was used for all efficacy analyses and included all participants who were randomized into the study and received at least one dose of study drug. The primary endpoint was analyzed using a repeated measures model, incorporating treatment group, smoking status, responsiveness to ipratropium bromide and/or albuterol, age (<65 years or ≥65 years) and sex as fixed effect class terms and a continuous covariate for baseline FEV_1_. A random effect for subject and a time effect (D15, D16, D28 and D29) were also included in the model. Least squares (LS) means and 95% confidence intervals (CIs) were calculated for the differences between each revefenacin dose and placebo on the primary endpoint, and statistical significance of pairwise comparisons were evaluated using the Benjamini-Hochberg step-up adjustment for multiple comparisons for the primary endpoint. A similar analysis methodology was used to assess the secondary spirometry endpoints and rescue medication use with no adjustment for multiplicity.

PK data were analyzed using summary statistics for all patients who provided PK data for at least one dose of revefenacin. Key PK parameters calculated included the time to maximum plasma concentration (t_max_), the maximum plasma concentration (C_max_), area under the (AUC) plasma concentration-time curve from time 0 to 24 h (AUC_0–24_) and from time 0 to the last detectable time point (AUC_0–t_) and terminal elimination half-life (t_1/2_).

The safety population was used for all safety analyses and included all subjects who were randomized into the study and received at least one dose of study drug.

## Results

### Patients

**Fig. 1:**
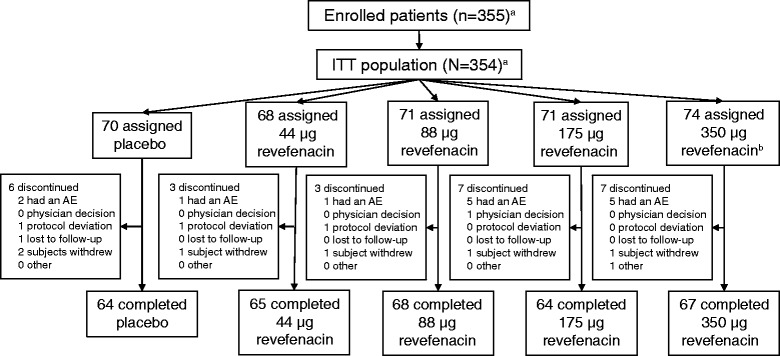
Patient disposition (ITT population). ^a^One patient, identified as participating in another clinical study during this study, was removed from all efficacy analyses (*n* = 354) but retained in all safety analyses (*n* = 355). ^b^One patient receiving revefenacin 350 μg was withdrawn from the study by the investigator on D6 due to a non-treatment–emergent prolonged QTcF (>500 msc) during the screening electrocardiogram. The subject is included in both the ITT and safety populations though the prolonged QTcF adverse event is not contained in the treatment-emergent AE summaries. Abbreviations: AE: adverse event; ITT: intent to treat; QTcF: Fridericia formula for QT interval correction

In total, 355 patients were enrolled in the study (Fig. 1). One patient, identified as participating in another clinical study, was removed from the ITT population (*N* = 354) but retained in the safety population (*N* = 355). One patient receiving revefenacin 350 μg was removed from the study on D6 due to a non-treatment–emergent prolonged Fridericia formula for QT interval correction (QTcF, >500 msc) revealed during the screening ECG but not reviewed by the investigator until post first dose. That subject is included in both the ITT and safety populations though the prolonged QTcF adverse event is not contained in the treatment-emergent AE summaries.

Patient demographics and key baseline characteristics, which were similar between treatment groups, are summarized in Table 1.

**Table 1:** Baseline demographics and clinical characteristics (ITT population)

		Revefenacin				
	Placebo (*n* = 70)	44 μg (*n* = 68)	88 μg (*n* = 71)	175 μg (*n* = 71)	350 μg (*n* = 74)	Total (*N* = 354)
Age (years)						
Mean (SD)	62.2 (9.17)	60.9 (8.87)	60.4 (7.98)	64.5 (7.69)	61.4 (8.98)	61.9 (8.63)
Sex (%)						
Male	37 (52.9)	32 (47.1)	32 (45.1)	37 (52.1)	40 (54.1)	178 (50.3)
Female	33 (47.1)	36 (52.9)	39 (54.9)	34 (47.9)	34 (45.9)	176 (49.7)
Race (%)						
White	65 (92.9)	63 (92.6)	64 (90.1)	65 (91.5)	67 (90.5)	324 (91.5)
Black/African American	4 (5.7)	4 (5.9)	6 (8.5)	5 (7.0)	6 (8.1)	25 (7.1)
Other	1 (1.4)	1 (1.5)	1 (1.4)	1 (1.4)	1 (1.4)	5 (1.4)
BMI (kg/m^2^)						
Mean (SD)	27.6 (5.73)	27.9 (6.87)	27.0 (5.92)	28.0 (5.30)	29.0 (5.76)	27.9 (5.93)
Current smoker (%)						
Yes	35 (50.0)	40 (58.8)	44 (62.0)	33 (46.5)	38 (51.4)	190 (53.7)
Smoking duration (pack-years)						
Mean (SD)	38.2 (9.71)	39.3 (11.59)	38.9 (10.16)	41.2 (10.01)	39.6 (9.04)	39.5 (10.11)
Current ICS use (%)						
Yes	29 (41.4)	25 (36.8)	24 (33.8)	26 (36.6)	26 (35.1)	130 (36.7)
Pre-dose FEV_1_ (mL)						
Mean (SD)	1205.0 (473.00)	1279.0 (470.10)	1321.0 (441.58)	1267.2 (415.67)	1338.8 (483.95)	1282.6 (457.25)
Pre-dose FEV_1_/FVC (%)						
n	70	68	71	71	72	352
Mean (SD)	0.50 (0.11)	0.51 (0.11)	0.52 (0.09)	0.51 (0.10)	0.52 (0.09)	0.51 (0.10)
Percent predicted post-bronchodilator FEV_1_ (%)						
n	70	68	71	71	72	352
Mean (SD)	41.2 (13.00)	43.0 (13.35)	45.1 (12.43)	44.0 (11.76)	44.5 (12.35)	43.6 (12.58)

*Abbreviations*: *BMI* body mass index, *FEV*_*1*_ forced expiratory volume in 1 s, *FVC* forced vital capacity, *ICS* inhaled corticosteroid, *ITT* intent to treat, *SD* standard deviation

### Efficacy

**Fig. 2:**
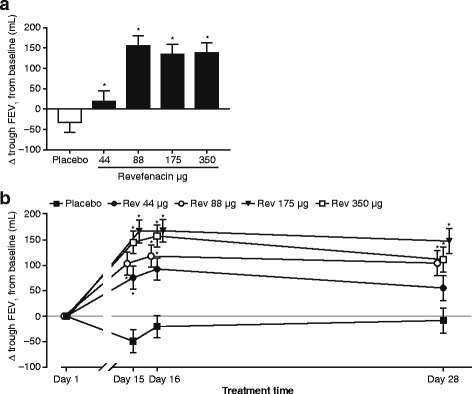
Change from baseline in trough FEV_1_. **a** D28^a^ trough FEV_1_ and **b** trough FEV_1_ on D1, D15, D16 and D28. Data presented as LS mean ± SE. *p < 0.001 versus placebo. Abbreviations: FEV_1:_ forced expiratory volume in 1 s; LS: least squares; Rev.: revefenacin; SE: standard error. ^a^D28 trough FEV_1_ measured on D29

The primary endpoint, change from baseline in D28 trough FEV_1_, demonstrated significant benefit with revefenacin over placebo for the three higher doses during the 24-h serial spirometry assessments (Table 2). On the D28 trough FEV_1_ measured on D29 (Fig. 2a), revefenacin 88 μg showed the largest improvement over placebo in trough FEV_1_ (LS mean [95% CI], 187.4 [118.8, 256.1] mL; *p* < 0.001 versus placebo) with the two higher doses also showing large improvements (166.6 [97.3, 263.0] mL; *p* < 0.0001 and 170.6 [101.9, 239.3] mL; *p* < 0.0001, for 175 and 350 μg, respectively). A non-statistically significant and likely sub-therapeutic improvement was noted for 44 μg (51.8 [−17.3, 121.0] mL).

**Table 2:** Summary of key spirometric measurements (ITT population)

		Revefenacin			
	Placebo	44 μg	88 μg	175 μg	350 μg
D28^a^ trough FEV_1_ change from baseline, mL					
n	55	60	63	59	63
LS mean (SE)	−32.4 (25.36)	19.4 (24.98)	155.0 (24.61)	134.2 (25.07)	138.2 (24.38)
LS mean difference from placebo (95% CI)	NA	51.8 (−17.3, 121.0)	187.4 (118.8, 256.1)**	166.6 (97.3, 236.0)**	170.6 (101.9, 239.3)**
D1 weighted mean FEV_1(0–6h),_ mL					
n	69	66	68	70	70
LS mean (SE)	33.3 (15.93)	145.1 (16.57)	193.8 (16.42)	180.0 (16.06)	214.9 (15.98)
LS mean difference from placebo (95% CI)	NA	111.7 (67.6, 155.9)**	160.5 (116.4, 204.5)**	146.7 (103.2, 190.2)**	181.5 (138.1, 225.0)**
D28 weighted mean FEV_1(0–12h),_ mL					
n	55	63	63	59	64
LS mean (SE)	−32.8 (27.03)	34.3 (26.13)	129.8 (26.10)	123.4 (26.88)	143.1 (25.42)
LS mean difference from placebo (95% CI)	NA	67.1 (−4.9, 139.2)	162.6 (90.5, 234.7)**	156.2 (83.0, 229.4)**	176.0 (104.3, 247.7)**
D28 weighted mean FEV_1(0–24h)_, mL					
n	54	59	63	59	62
LS mean (SE)	−78.1 (25.46)	3.4 (25.21)	87.0 (24.50)	84.1 (24.97)	96.3 (24.13)
LS mean difference from placebo (95% CI)	NA	81.5 (12.8, 150.2)*	165.1 (97.2, 233.0)**	162.1 (93.4, 230.8)**	174.3 (106.5, 242.2)**

* *p* = 0.02, **p < 0.001 versus placebo

*Abbreviations*: *CI* confidence interval, *FEV*_*1*_ forced expiratory volume in 1 s, *FEV*_*1(0–6)*_ FEV_1_ over 0 to 6 h, *FEV*_*1(0–12)*_ FEV_1_ over 0 to 12 h, *FEV*_*1(0–24)*_ FEV_1_ over 0 to 24 h_,_ *ITT* intent-to-treat, *LS* least squares, *NA* not applicable, *SE* standard error

^a^D28 trough FEV_1_ measured on D29

On prior trough FEV_1_ assessments (D15, D16 and D28), revefenacin 175 μg consistently outperformed the other two therapeutic doses (Fig. 2b) with mean additional improvements in FEV_1_ ranging from 25.7 to 58.1 mL relative to 88 μg and ranging from 14.3 to 27.9 mL relative to 350 μg. All three higher doses produced improvements in trough FEV_1_ exceeding 100 mL during the 28 days of follow up, while the placebo group showed small declines.

**Fig. 3:**
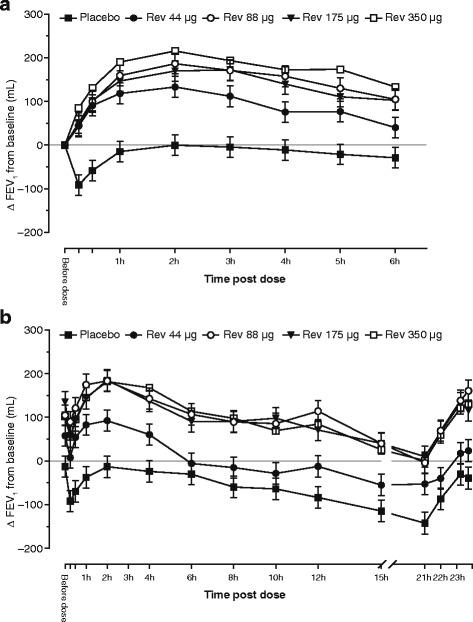
Change from baseline in serial FEV_1_ on Day 1 (**a**) and Day 28 (**b**). Data presented as LS mean ± SE. Abbreviations: FEV_1_: forced expiratory volume in 1 s; h: hours; LS: least squares, Rev.: revefenacin; SE: standard error

Revefenacin demonstrated a rapid onset of brochodilation at all doses on D1 (Fig. 3). The median (95% CI) time to achieve at least a 100-mL FEV_1_ increase from baseline on D1 was 30 (30, 60) minutes for both 88 and 175 μg and 30 (15, 30) minutes for 350 μg (nominal *p* < 0.001 versus placebo for all doses). At doses ≥88 μg, more than 80% of patients achieved at least a 100-mL FEV_1_ increase from baseline in the first 4 h post dose compared with 33% of placebo patients. The three higher revefenacin doses showed mean improvements over placebo that exceeded 114 mL and were present by 1 h post dose on D1 and throughout all timepoints on D28 (Fig. 3). The D1 weighted mean FEV_1(0–6h)_ ranged from 145.1 mL (44 μg) to 214.9 mL (350 μg), with significant increases versus placebo noted for all doses (Table 2). Additionally, for doses ≥88 μg on D28, differences from placebo for both weighted mean FEV_1(0–12h)_ and FEV_1(0–24h)_ values were numerically similar to respective trough FEV_1_ values, suggesting sustained 24-h bronchodilation.

**Fig. 4:**
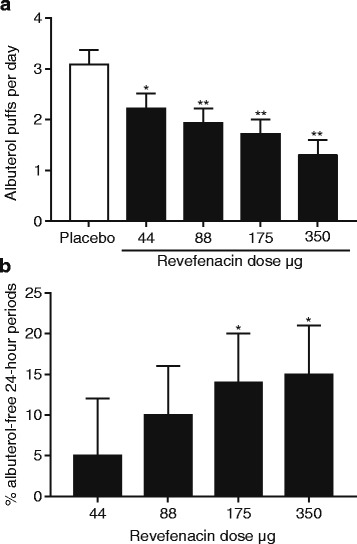
Effect of revefenacin on rescue medication use. **a** albuterol puffs per day; data presented as LS mean ± SE. **b** percent of albuterol-free 24-h periods over the entire treatment period; data presented as LS mean difference from placebo ± SE. *p < 0.05; **p < 0.005. Abbreviations: LS: least squares; SE: standard error

Revefenacin decreased albuterol rescue inhaler use compared with placebo for the three highest doses. On average, placebo subjects used three puffs/day of rescue albuterol (Fig. 4a). Doses ≥88 μg significantly decreased rescue medication use by a clinically relevant decrease of at least one puff per day in a dose-dependent manner (*p* < 0.005). Expressed as rescue inhaler-free days over the entire treatment period, revefenacin 175 μg and 350 μg increased the number of rescue-free days over placebo by ≥14% (*p* < 0.05; Fig. 4b), corresponding to an increase of approximately one rescue-free day per week.

**Fig. 5:**
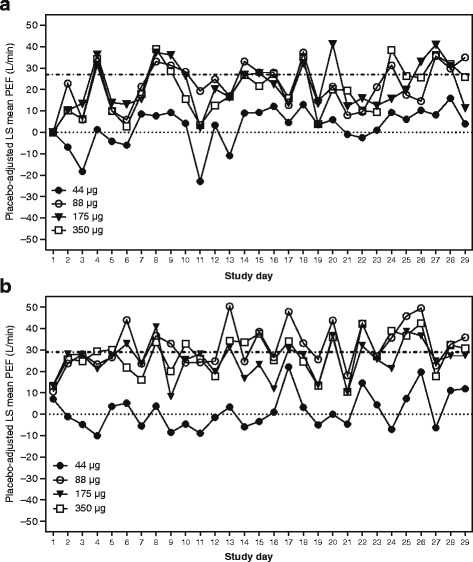
Effect of revefenacin on daily peak expiratory flow rate. **a** morning PEF (representative of the trough PEF of the prior day’s dosing). **b** evening PEF, assessed ≈12 h after daily study medication dosing. Data presented as placebo-adjusted LS mean; dotted line (·········) = zero L/min; dashed line (−·­·­·­·­) represents LS mean placebo–adjusted increases in AM and PM PEF for revefenacin doses ≥88 μg, which were similar across the treatment period and centered at approximately 27 L/min and 29 L/min, respectively. Abbreviations: LS: least squares; PEFR: peak expiratory flow rate

Throughout the 28-day study period, revefenacin produced sustained increases in morning and evening PEF (Fig. 5); PEF measurements reached steady state early in dosing, prior to D7, and were maintained throughout the remaining evaluation period. For revefenacin doses ≥88 μg, LS mean placebo-adjusted increases in AM and PM PEF showed clinically relevant improvements of approximately 27 L/min and 29 L/min, respectively. In contrast, the PEF response for revefenacin 44 μg fell below that of the other doses for the majority of the evaluation period.

### Pharmacokinetics

Plasma concentration data were received and analyzed for 34 subjects treated with revefenacin 44, 88, 175 or 350 μg for 28 days. Median t_max_ values ranged from 0.483 to 0.517 h and mean C_max_ values ranged from 0.0203 to 0.146 ng/mL (Table 3). After reaching maximum concentrations, revefenacin concentrations declined rapidly in a biphasic manner and were followed by a slow apparent terminal elimination phase characterized by mean t_1/2_ values of 51.9 and 57.9 h at the 175- and 350-μg dose levels, respectively. Mean AUC_0–24_ values ranged from 0.0347 to 0.365 ng•hr./mL, and mean AUC_0–t_ values ranged from 0.0299 to 0.724 ng•hr./mL. Relative to the 44 μg dose, mean C_max_ and AUC values increased with the increases in dose level. Conclusions with respect to dose proportionality were not drawn in this study due to the high variability and limited sample size in each dose group.

**Table 3:** Summary of mean plasma pharmacokinetic parameters^a^

Dose (μg)	C_max_ (ng/mL)	t_max_ (hr)	AUC_0-24_ (ng●hr./mL)	AUC_0–t_ (ng●hr./mL)	t_1/2_ (hr)
44	0.0203 ± 0.0124	0.483 (0.350, 0.500)	0.0347 ± 0.0106	0.0299 ± 0.0098	NC^b^
n	7	7	4	4	1
88	0.0282 ± 0.0157	0.500 (0.417, 12.3^c^)	0.155 ± 0.0749	0.129 ± 0.0589	NC
n	9	9	6	6	0
175	0.121 ± 0.157	0.517 (0.467, 0.767)	0.351 ± 0.364	0.720 ± 0.864	51.9 ± 31.5
n	9	9	9	9	5
350	0.146 ± 0.148	0.500 (0.433, 0.583)	0.365 ± 0.306	0.724 ± 0.833	57.9 ± 31.5
n	8	8	8	8	7

^a^Data are mean ± SD except for t_max_ data which are median (minimum, maximum)

^b^T_1/2_ values were not calculated for the majority of subjects at revefenacin 44 and 88 μg due to limited data at these dose levels

^c^The concentration observed at this t_max_ value was considered to be high compared to the rest of the subject’s profile and was reanalyzed. Reanalysis confirmed the original result, and the value was included in PK analysis

*Abbreviations*: *AUC*_*0–24*_ area under the curve for plasma concentration-time from time 0 to 24 h, *AUC*_*0–t*_ area under the curve for plasma concentration-time from time 0 to the last detectable time point, *C*_*max*_ maximum plasma concentration, *hr.* hours, *NC* not calculated, *PK* pharmacokinetics, *SD* standard deviation, *t*_*1/2*_ terminal elimination half-life, *t*_*max*_ time to maximum plasma concentration

### Safety

Of the 355 patients in the safety population, 328 (92.4%) completed the study per protocol. Twelve of 355 subjects (3.4%) had an AE that led to permanent treatment discontinuation, which occurred more frequently in the two highest-dose revefenacin treatment groups (7.0% [*n* = 71] and 5.4% [*n* = 74] in the 175 μg and 350 μg groups, respectively) than in the other treatment groups (1.4% [*n* = 71], 1.5% [*n* = 68] and 1.4% [*n* = 71] in the placebo and revefenacin 44 μg and 88 μg groups, respectively). With the exception of one event of respiratory distress experienced by a placebo patient, all events resolved.

Revefenacin was generally well tolerated, and the frequency of AEs was broadly comparable across all treatment groups, including placebo. Headache, shortness of breath and cough were the three most common AEs in the study (Table 4). A small number of treated subjects (five of 355) experienced worsening of COPD (four exacerbations and one worsening of symptoms) during the course of the study; these subjects were withdrawn from the study and treated appropriately.

**Table 4:** Treatment-emergent AEs reported in ≥1% of any treatment group (safety population)

		Revefenacin				
Patients with an AE, *n* (%)	Placebo (*n* = 71)	44 μg (*n* = 68)	88 μg (*n* = 71)	175 μg (*n* = 71)	350 μg (*n* = 74)	Total (*N* = 355)
Any AEs	22 (31.0)	16 (23.5)	26 (36.6)	22 (31.0)	23 (31.1)	109 (30.7)
Preferred term						
Headache	2 (2.8)	1 (1.5)	2 (2.8)	1 (1.4)	5 (6.8)	11 (3.1)
Dyspnea	2 (2.8)	0	3 (4.2)	3 (4.2)	2 (2.7)	10 (2.8)
Cough	1 (1.4)	0	0	3 (4.2)	3 (4.1)	7 (2.0)
COPD^a^	2 (2.8)	0	0	1 (1.4)	2 (2.7)	5 (1.4)
Back pain	0	0	1 (1.4)	2 (2.8)	1 (1.4)	4 (1.1)
Oropharyngeal pain	1 (1.4)	1 (1.5)	0	0	2 (2.7)	4 (1.1)

^a^This MedDRA PT is used in the event that a patient’s COPD worsens

*Abbreviations*: *AE* adverse event, *COPD* chronic obstructive pulmonary disease, *MedDRA* Medical Dictionary for Regulatory Activities, *PT* preferred term

Four SAEs were reported during the study; one in the 44 μg group (supraventricular tachycardia), two in the 175 μg group (unstable angina and hypertension) and one in the 350 μg group (intestinal obstruction). One of the four SAEs (supraventricular tachycardia) was considered by the blinded investigator to be possibly related to revefenacin, but an independent assessment by a blinded cardiologist confirmed that the condition was present during screening, the change following dosing was minimal and the likelihood of a relationship between this SAE and the study medication was low. All four SAEs resolved, but the subjects with cases of supraventricular tachycardia and unstable angina were withdrawn from the study.

AEs traditionally associated with inhaled anti-muscarinic agents were infrequent. Two incidents of dry mouth (one each in the 88 μg and 350 μg groups; both mild in severity) were observed, but no incidents of constipation, urinary retention, dysuria, blurred vision or glaucoma were reported.

Holter monitoring findings were unremarkable for changes of mean, maximum or minimum heart rates during the recording period, with no findings that were uniquely associated with revefenacin dosing or that were clearly more prominent with increasing revefenacin dose level.

## Discussion

This study characterized the efficacy and safety of revefenacin (44, 88, 175 or 350 μg) after 28 days of once-daily administration to patients with moderate to severe COPD. Revefenacin at doses ≥88 μg led to significant improvements in the primary endpoint (change from baseline in D28 trough FEV_1_). The trough FEV_1_ improvements with the 88 and 175 μg dose (155 and 134 mL, respectively) are comparable to reported values for tiotropium bromide (126 mL) [21] delivered with the Respimat® soft mist inhaler.

As a novel once-daily LAMA, revefenacin may be differentiated from the currently available once-daily tiotropium and umeclidinium handheld products in important ways. Revefenacin’s novel biphenyl carbamate tertiatry amine structure is distinct from the quaternary ammonium antagonists, [22] thus representing the first inhaled LAMA of its class to enter clinical development for COPD. The revefenacin discovery program was designed to select a 24-h–duration LAMA endowed with chemical stability (enabling long-term storage as a preservative-free aqueous solution product), high lung-to-salivary gland functional selectivity (vida infra) and a metabolically labile primary amide “soft-drug” site to allow rapid systemic clearance of the parent drug, thus potentially minimizing systemically mediated adverse events [16].

The high metabolic lability of revefenacin contrasts with the relative metabolic stability of tiotropium and its primarily renal systemic clearance profile. Instead, revefenacin shares umeclidinium’s profile of rapid metabolic turnover after distributing from the lung [23]. Systemic clearance of revefenacin is primarily via enzymatic hydrolysis versus cytochrome P450 (CYP2D6)-mediated oxidative turnover for umeclidinium [24]. Finally, the revefenacin drug product in the current study is administered via a standard jet nebulizer. This unique presentation of a once-daily bronchodilator may be of future therapeutic benefit to those patients who prefer or require nebulization therapy [9].

The sustained 24-h bronchodilator response observed previously in a 7-day study of revefenacin in patients with moderate to severe COPD [17] was supported here by the numerical similarity of trough FEV_1_ values to the D1 and D28 weighted mean FEV_1_ values. A sustained response was further supported by the observation of increased morning and evening PEFs throughout the treatment period, which is consistent with reports of once-daily tiotropium following 3 or 4 weeks of dosing in patients with moderate to severe COPD [21, 25]. In addition, patients treated with revefenacin used less rescue albuterol throughout the 4-week treatment period, in keeping with reduced rescue medication use reported for tiotropium [21] following 4 and 12 weeks of dosing.

Revefenacin was generally well tolerated and did not produce systemic effects typically associated with anti-cholinergic therapies, such as dry mouth, urinary retention, tachycardia or acute closed-angle glaucoma [26]. The incidence of dry mouth (1.4% for both 175 and 350 μg) was lower than the 4% incidence reported in a comparable 3-week study of 5 μg tiotropium administered with the Respimat soft mist inhaler in patients with COPD [25]. In addition, the revefenacin safety profile observed here was consistent with pre-clinical studies that revealed a superior lung selectivity index (ratio of anti-sialagogue to bronchoprotective potency) with inhaled revefenacin compared with either glycopyrronium or tiotropium [27]. Moreover, the favorable AE profile of revefenacin in this study is supported by a 7-day multiple-dose study of revefenacin (22 to 350 μg) in patients with moderate to severe COPD [17] in which the frequency of AEs was comparable across all treatment groups, including placebo.

The efficacy and safety data from this study indicate that revefenacin 88 and 175 μg represent apppropriate doses for use in longer-term safety and efficacy trials. The trough FEV_1_ data indicated that 44 μg was a sub-therapeutic revefenacin dose compared with the three higher doses. Additionally, the 350 μg dose did not demonstrate additional efficacy over that observed with 175 μg revefenacin. Limitations of this study include the small number of patients and the relatively short 4-week treatment period, which limit conclusions regarding the potential clinical benefits of revefenacin. However, the data from this study have informed dose selection for longer-term phase 3 clinical trials investigating the safety [15] and efficacy [14] of revefenacin in patients with moderate to very severe COPD. The results of those studies may reveal the potential clinical benefits of revefenacin in patients with COPD.

More broadly, certain COPD patient populations may especially benefit from the use of nebulizer therapy, such as those with chronic muscle weakness, the elderly and those with cognitive or visual impairment or diminished manual dexterity [6]. In general, these patients also prefer nebulized therapy when hospitalized for exacerbations of COPD [23]. Currently these patients are managed with nebulized twice-daily long-acting beta-agonists (arformoterol tartrate and formoterol fumarate) or short-acting nebulized agents requiring four-times a day dosing. Revefenacin, if approved, could offer patients who require or prefer nebulized therapy the opportunity to be treated with a once-daily LAMA, as has been standard practice for many years in COPD patients who can use handheld devices containing the once-daily LAMAs (including tiotropium and umeclidinium) [22]. Additionally, revefenacin would represent the first once-daily LAMA approved for nebulized therapy, with similar efficacy to tiotropium but with less potential for antimuscarinic side effects (eg, dry mouth) [9, 28].

## Conclusion

Once-daily revefenacin increased trough FEV_1_ in patients with moderate to severe COPD, with 88 and 175 μg revefenacin identified as the optimal doses for use in longer-term safety and efficacy studies. Revefenacin has the potential to be the first once-daily long-acting bronchodilator for use in patients who require or prefer nebulized anti-muscarinic therapy.

## Abbreviations

AE: Adverse event
AUC: Area under the curve
AUC_0–24_: AUC for plasma concentration-time from time 0 to 24 h
AUC_0–t_: AUC for plasma concentration-time from time 0 to to the last detectable time point
CI: Confidence interval
C_max_: Maximum plasma concentration
COPD: Chronic obstructive pulmonary disease
ECG: Electrocardiogram
FEV_1_: Forced expiratory volume in 1 s
FEV_1(0–12)_: Forced expiratory volume in 1 s over 0 to 12 h
FEV_1(0–24)_: Forced expiratory volume in 1 s over 0 to 24 h
FEV_1(0–6)_: Forced expiratory volume in 1 s over 0 to 6 h
ICS: Inhaled corticosteroids
ITT: Intent to treat
LABA: Long-acting beta agonist
LAMA: Long-acting muscarinic antagonist
LS: Least squares
MedDRA: Medical Dictionary for Regulatory Activities
NC: Not calculated
PEF: Peak expiratory flow
PK: Pharmacokinetic
PT: Preferred term
QTcF: Fridericia formula for QT interval correction
REV: Revefenacin
SAE: Serious adverse event
SD: Standard deviation
SE: Standard error
t_1/2_: Terminal elimination half-life
t_max_: Time to maximum plasma concentration

## Footnotes

1 A preliminary abstract of this study was previously published: Nicholls AJ, Barnes C, Bourdet DL, Moran EJ, Haumann B. Dose-Ranging Study Of Once-Daily TD-4208, An Inhaled Long-Acting Muscarinic Antagonist (LAMA) In Patients With Chronic Obstructive Pulmonary Disease (COPD). Am J Respir Crit Care Med. 2015;191:A5750 [Abstract].
